# 380. Implementing the Comparing Oral versus Parenteral Antimicrobial Therapy (COPAT) Clinical Trial to Influence Institutional Practice Transformation Towards Earlier Transition to Oral Antibiotics

**DOI:** 10.1093/ofid/ofaf695.127

**Published:** 2026-01-11

**Authors:** Joy J Juskowich, Jesse M Thompson, Seyoum D Bage, Karen M Palmateer, John A Guilfoose, Allison Lastinger, Connie L Smith, Victor A Arcega, Jonathan E Stanley, Haley M Summerfield, Cynthia Fisher-Duda, Manoj Nepal, Arif R Sarwari

**Affiliations:** West Virginia University, Morgantown, West Virginia; West Virginia University, Morgantown, West Virginia; WVU Medicine, Parkersburg, West Virginia; WVU Medicine, Parkersburg, West Virginia; West Virginia University, Morgantown, West Virginia; West Virginia University Hospital, Morgantown, West Virginia; West Virginia University, Morgantown, West Virginia; West Virginia University, Morgantown, West Virginia; WVU Medicine, Parkersburg, West Virginia; WVU Medicine, Parkersburg, West Virginia; West Virginia University, Morgantown, West Virginia; West Virginia University, Morgantown, West Virginia; West Virginia University, Morgantown, West Virginia

## Abstract

**Background:**

Two landmark clinical trials in 2019 established the scientific evidence behind early oral (PO) antibiotic transition. However, this has yet to be widely accepted and Outpatient Parenteral Antimicrobial Therapy (OPAT) programs have proliferated. Complex Outpatient Antimicrobial Therapy with Oral Agents (COpAT) programs face significant implementation science challenges. We executed a clinical trial to influence practice transformation toward earlier PO antibiotic transition and accelerate our COpAT program growth.

**Figure:**
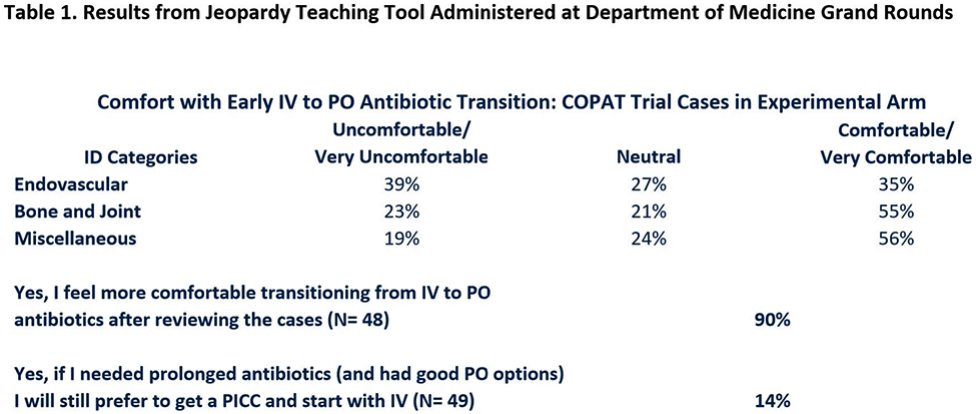

**Figure:**
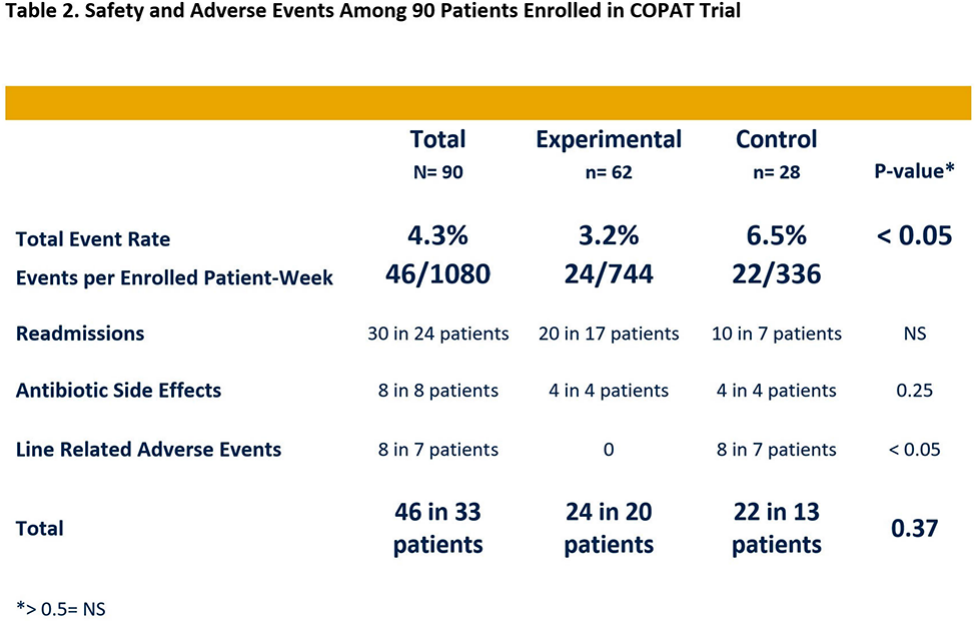

**Methods:**

Comparing Oral vs. Parenteral Antimicrobial Therapy (COPAT) Trial was a pragmatic, randomized controlled trial at five hospitals in one health system. Patients being discharged with two or more weeks of intravenous (IV) antibiotics were randomized 2:1 to early PO (*Experimental*) vs. continued IV (*Control*). Two primary outcomes were assessed at three months: *Experimental* group safety superiority and Efficacy equivalence. A Jeopardy teaching tool using COPAT Trial cases was presented at multiple forums. Providers recorded their baseline discomfort with early PO antibiotic transition and noted whether the presentation helped (Table 1). The trial was stopped at 2/3 enrollment (*Experimental* 62, *Control* 28) based on a significant safety benefit.

**Figure:**
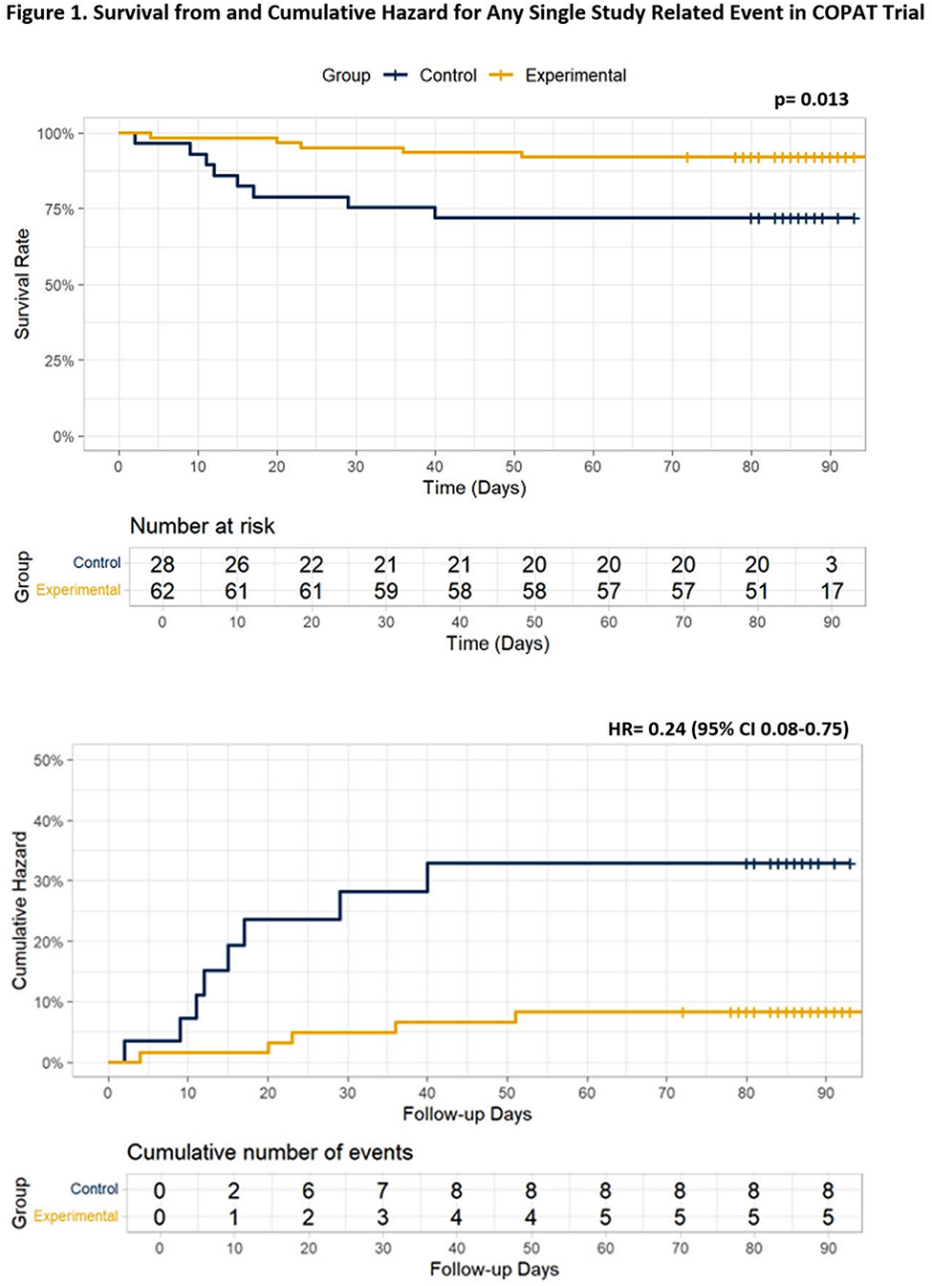

**Figure:**
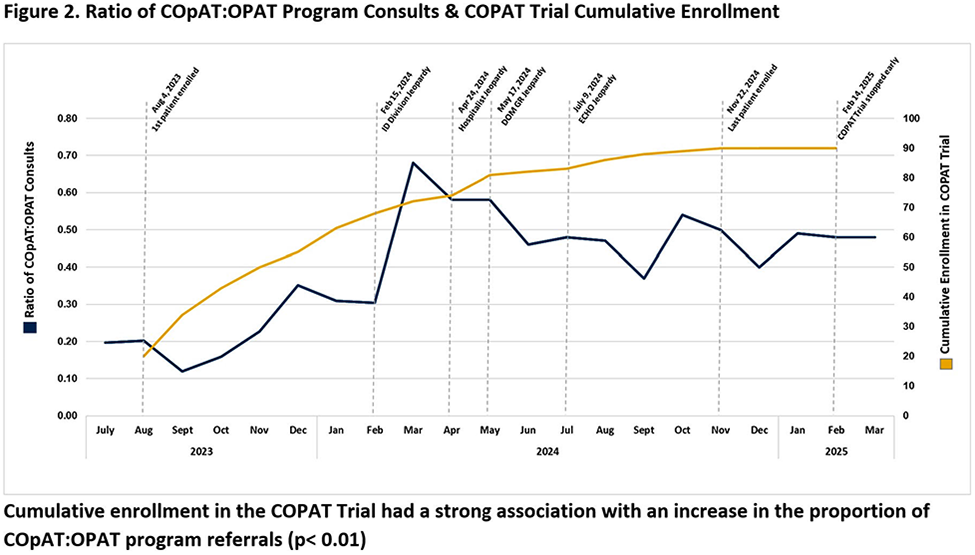

**Results:**

Both groups had similar background characteristics with a predominance of bone/joint (73%) followed by endovascular infections (14%). Total antibiotic duration was equivalent. PO antibiotic transition occurred at median four days. *Experimental* group demonstrated a significantly lower adverse event rate (*Experimental* vs. *Control*: 3.2% vs. 6.5%, p< 0.05; Table 2). Survival from and cumulative hazard for any single study related event were both significant with curves separating by day 10 (Figure 1). Treatment efficacy was equivalent in both groups. COpAT:OPAT program consult ratio increased as the teaching tool was implemented (Figure 2). Also, as provider comfort with earlier PO antibiotic transition improved, COPAT Trial enrollment rate decreased.

**Conclusion:**

Early IV to PO antibiotic transition was significantly safer and did not compromise clinical outcomes. At our academic health system, implementation of the COPAT Trial with case-based education accelerated practice transformation.

**Disclosures:**

All Authors: No reported disclosures

